# Resonance Spectrum Characteristics of Effective Electromechanical Coupling Coefficient of High-Overtone Bulk Acoustic Resonator

**DOI:** 10.3390/mi7090159

**Published:** 2016-09-06

**Authors:** Jian Li, Mengwei Liu, Chenghao Wang

**Affiliations:** 1 Institute of Acoustics, Chinese Academy of Sciences, Beijing 100190, China; lijian212@mails.ucas.ac.cn; 2 University of Chinese Academy of Sciences, Beijing 100049, China

**Keywords:** high-overtone bulk acoustic resonator, effective electromechanical coupling coefficient, resonance spectrum

## Abstract

A high-overtone bulk acoustic resonator (HBAR) consisting of a piezoelectric film with two electrodes on a substrate exhibits a high quality factor (*Q*) and multi-mode resonance spectrum. By analyzing the influences of each layer’s material and structure (thickness) parameters on the effective electromechanical coupling coefficient (*K*_eff_^2^), the resonance spectrum characteristics of *K*_eff_^2^ have been investigated systematically, and the optimal design of HBAR has been provided. Besides, a device, corresponding to one of the theoretical cases studied, is fabricated and evaluated. The experimental results are basically consistent with the theoretical results. Finally, the effects of *K*_eff_^2^ on the function of the crystal oscillators constructed with HBARs are proposed. The crystal oscillators can operate in more modes and have a larger frequency hopping bandwidth by using the HBARs with a larger *K*_eff_^2^·*Q*.

## 1. Introduction

**Figure 1:**
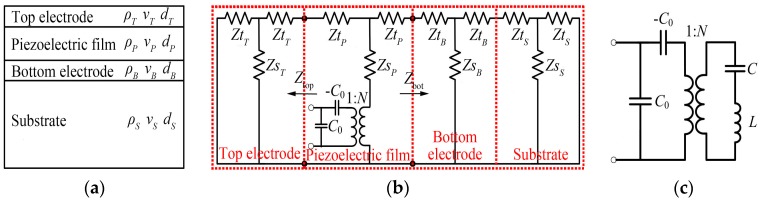
(**a**) The cross-section schematic of HBAR; (**b**) The one-dimensional Mason equivalent circuit model; (**c**) The lumped parameter equivalent circuit near the resonance frequency.

The high-overtone bulk acoustic resonator (HBAR) structure consists of a piezoelectric sandwich micro-transducer and a substrate with low acoustic attenuation, where the transducer is composed of a piezoelectric film, a top electrode and a bottom electrode, as shown in Figure 1a. The acoustic wave excited by the thickness vibration of an electrically loaded piezoelectric plate propagates into the substrate, which will lead to the standing wave resonance. Since the substrate thickness is much larger than the acoustic wavelength, the HBAR will operate at a number of resonance frequencies; namely, HBAR has multi-frequency resonance characteristics, and it can be used as a multiple-frequency microwave source [1] and an agile-frequency synthesizer in electronic warfare. Besides, HBAR has a large quality factor (*Q*) due to the low acoustic attenuation substrate, and exhibits the largest *Q*-Frequency product of the crystal oscillators. Thus, it can also be applied in the low-phase noise microwave signal sources [2,3].

Zhang et al. [4,5,6] developed the resonance spectrum method for HBARs to study the resonance spectrum, the variations of the effective electromechanical coupling coefficient (*K*_eff_^2^) and frequency interval with the resonance spectrum, and the effects of the electrode thickness on *K*_eff_^2^. Pao et al. [7] discussed the variations of *K*_eff_^2^ with the substrate thickness for the HBAR with different substrate materials. Zhang et al. [8,9,10,11,12] conducted more in-depth research on the effects of the electrode and substrate materials as well as their thickness on the resonance spectrum distribution and the corresponding *K*_eff_^2^. These above studies have mainly focused on *K*_eff_^2^-frequency distribution of the HBAR, and the effects of each layer’s thickness on *K*_eff_^2^ have been only carried out in a special case, i.e., the so-called normal region. In our recent work [13], the influences of the continuous change of the thickness of several substrates, piezoelectric films and electrodes on *K*_eff_^2^ have been investigated systematically.

This paper presents a study on the resonance spectrum characteristics of *K*_eff_^2^ changing with several common substrates, electrodes and piezoelectric films as well as their thickness. Besides, a device, corresponding to one of the theoretical cases studied, is fabricated and evaluated. Finally, the effects of *K*_eff_^2^ on the function of the crystal oscillators with HBARs are discussed. Based on this study, a set of guidelines for the optimal selection of the HBAR’s parameters are provided.

## 2. The Lumped Parameter Equivalent Circuit of HBAR

The characteristics of HBAR can be analyzed by a one-dimensional Mason equivalent circuit [14], shown in Figure 1b, where *Zt_T_* = *jZ_T_*tan(*α_T_*/2), *Zt_P_* = *jZ_P_*tan(*α_P_*/2), *Zt_B_* = *jZ_B_*tan(*α_B_*/2) and *Zt_S_* = *jZ_S_*tan(*α_S_*/2); *Zs_T_* = *Z_T_*/(*j*sin*α_T_*), *Zs_P_* = *Z_P_*/(*j*sin*α_P_*), *Zs_B_* = *Z_B_*/(*j*sin*α_B_*) and *Zs_S_* = *Z_S_*/(*j*sin*α_S_*). *Z_T_* = *ρ_T_ν_T_D_T_*, *Z_P_* = *ρ_P_ν_P_D_P_*, *Z_B_* = *ρ_B_ν_B_D_B_* and *Z_S_* = *ρ_S_ν_S_D_S_* are the characteristic acoustic impedances. *α_T_* = *k_T_d_T_*, *α_P_* = *k_P_d_P_*, *α_B_* = *k_B_d_B_* and *α_S_* = *k_S_d_S_*; *k_T_* = *ω_T_*/*ν_T_*, *k_P_* = *ω_P_*/*ν_P_*, *k_B_* = *ω_B_*/*ν_B_* and *k_S_* = *ω_S_*/*ν_S_* are the wave vectors, and the subscripts *T*, *P*, *B*, *S* represent the top electrode, piezoelectric film, bottom electrode and substrate, respectively. *ρ*, *ν*, *d* are the density, extensional wave velocity and thickness, as shown in Figure 1a. *D* is the active area of the HBAR; *ω* is the angular frequency of the resonator; *C*_0_ = *ε*_33_^S^*D*/*d_P_* is the static capacitance of the resonator; *N*^2^ = *C*_0_^2^*k_t_*^2^*c*_33_^D^/*ε*_33_^S^ is the electromechanical conversion ratio; and *ε*_33_^S^, *c*_33_^D^ and *k_t_*^2^ are the permittivity at constant strain, the stiffness coefficient at constant electric displacement and the thickness extensional electromechanical coupling coefficient of the piezoelectric film, respectively.

The parallel resonance frequency equation is

(1) $${Z_{P}}^{2}+Z_{\mathrm{top}}Z_{\mathrm{bot}}-jZ_{P}\left(Z_{\mathrm{top}}+Z_{\mathrm{bot}}\right)\cot\alpha_{P}=0$$

*Z*_top_ and *Z*_bot_ are the input impedances on both sides of the piezoelectric layer, which are given by:

(2) $$Z_{\mathrm{top}}=jZ_{T}\tan\alpha_{T},\;Z_{\mathrm{bot}}=jZ_{B}\frac{Z_{B}\tan\alpha_{B}+Z_{S}\tan\alpha_{S}}{Z_{B}-Z_{S}\tan\alpha_{B}\tan\alpha_{S}}$$

Equation (1) has multiple roots, which constitutes the resonance spectrum of a HBAR; the lower resonance frequency corresponds to a lower number of modes among the modes {*m*} = 1, 2, 3, ... *m*, *m* + 1, ... of a HBAR.

The distributed parameter equivalent circuit (see Figure 1b) can be simplified into the lumped parameter equivalent circuit (see Figure 1c) by the frequency shift method [15] near the concerned resonance frequencies. The mechanical compliance *C* and mechanical mass *L* of mode *m* are given by:

(3) $$C^{\left(m\right)}=\frac{2A_{2}}{\omega_{p}Z_{P}A_{1}},\;L^{\left(m\right)}=\frac{Z_{P}A_{1}}{2\omega_{p}A_{2}},$$

where

(4) $$\begin{array}{l} A_{1}=(Z_{P}-jZ_{\mathrm{top}}\cot\alpha_{P})[\frac{Z_{S}\tan\alpha_{B}+Z_{B}\tan\alpha_{S}}{Z_{B}-Z_{S}\tan\alpha_{B}\tan\alpha_{S}}\alpha_{S}-jZ_{\mathrm{bot}}(\frac{\alpha_{B}}{Z_{B}}+\frac{\alpha_{P}}{Z_{P}})]\\ -(\cot\alpha_{P}+j\frac{Z_{\mathrm{top}}}{Z_{P}})(Z_{B}\alpha_{B}+Z_{P}\alpha_{P}+\frac{Z_{S}-Z_{B}\tan\alpha_{B}\tan\alpha_{S}}{Z_{B}-Z_{S}\tan\alpha_{B}\tan\alpha_{S}}Z_{B}\alpha_{S})\\ -\alpha_{T}[jZ_{\mathrm{bot}}\frac{Z_{T}}{Z_{P}}-jZ_{\mathrm{top}}\frac{Z_{P}}{Z_{T}}+(\frac{Z_{\mathrm{top}}Z_{\mathrm{bot}}}{Z_{T}}-Z_{T})\cot\alpha_{P}] \end{array}$$

(5) $$A_{2}=2Z_{P}\tan(\alpha_{P}/2)-j(Z_{\mathrm{top}}+Z_{\mathrm{bot}})$$

The parallel and series resonance frequency of mode *m* are

(6) $$f_{p}^{\left(m\right)}=\frac{1}{2\pi \sqrt{L^{\left(m\right)}C^{\left(m\right)}}},\;f_{s}^{\left(m\right)}=f_{p}^{\left(m\right)}\sqrt{1-N^{2}\frac{C^{\left(m\right)}}{C_{0}}}$$

The *K*_eff_^2^ of mode *m* can be calculated as follows:

(7) $$K_{\mathrm{eff}}^{(m)2}=\frac{\pi^{2}}{8}\frac{f_{p}^{(m)2}-f_{s}^{(m)2}}{f_{p}^{(m)2}}=\frac{\pi^{2}}{8}N^{2}\frac{C^{\left(m\right)}}{C_{0}}=\frac{\pi^{2}{k_{t}}^{2}}{4\alpha_{P}}\frac{A_{2}}{A_{1}}$$

The superscript *m* of the parameters above is ignored in the following discussions for simplicity. The case ignoring the influence of the thin electrodes, i.e., *d_T_* = *d_B_*→0, will be discussed in Section 3 and Section 4, firstly, and the case considering the influence of the electrodes will be studied in Section 5. The material parameters used in the paper are listed in Table 1 [16,17].

## 3. Relationship between the Effective Electromechanical Coupling Coefficient and the Substrate

### 3.1. K_eff_^2^ Varies with the Substrate Thickness

**Figure 2:**
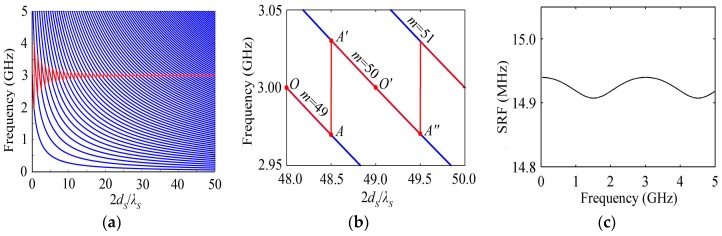
The HBAR consisting of ZnO_1.05μm_/Sapphire; (**a**) The variation curve of the resonance frequency with the substrate thickness; (**b**) The partial enlarged drawing of Figure 2a; (**c**) The change of SRF with the frequency when the substrate thickness 2*d_S_*/*λ_S_* = 200.

The blue lines of Figure 2a show the behavior of the resonance frequency, i.e., the resonance spectrum, with the normalized substrate thickness 2*d_S_*/*λ_S_* for the HBAR consisting of ZnO_1.05μm_/Sapphire, where *d_P_* = 1.05 μm = *λ_P_*/2, *λ_P_* and *λ_S_* are the wavelengths of piezoelectric film and substrate at 3 GHz, respectively, similarly hereinafter. The resonance spectrum is composed of a set of separated curves, and the different curves are corresponding to the different number of modes. The resonance frequencies are a series of discrete values for the HBAR with a certain substrate thickness, which forms the resonance spectrum with multi-resonance frequencies. The substrate thickness brings about a great difference of the resonance spectrum distribution; the frequency of the same number of modes and spacing of the resonance frequencies (SRF) are smaller for thicker substrates. Besides, with the increase of the resonance frequency, the SRF is fluctuating slightly rather than being a constant value, as shown in Figure 2c.

The case discussed in the normal region [4,5,6,7,8,9,10,11,12] is that the thickness of the piezoelectric film and substrate are a half-wavelength and an integer multiple of the half-wavelength at the resonance frequency *f*_0_, respectively, e.g., the case of the intersections of *f*_0_ and the resonance spectrum shown in blue lines of Figure 2a when *f*_0_ = 3 GHz. The case where the substrate thickness does not equal an integer multiple of the half-wavelength, i.e., the substrate thickness is between any two adjacent discrete values, has not been studied yet; the HBAR will not resonate at *f*_0_ (3 GHz) in such a situation. The behavior of the resonance frequency closest to *f*_0_ with the substrate thickness is shown in the red line of Figure 2a; for its partial enlarged drawing Figure 2b, the resonance frequency closest to *f*_0_ declines along the OA line with the increase of the substrate thickness, and then it jumps to the A′ point with the corresponding number of modes changing from 49 to 50 when 2*d_S_*/*λ_S_* ≈ 48.5. Similarly, the number of modes jumps to 51 at the A″ point in which 2*d_S_*/*λ_S_* ≈ 49.5. The red line in Figure 2a shows that the resonance frequency closest to *f*_0_ oscillates around *f*_0_, and the oscillation amplitude is smaller for thicker substrates. Actually, the HBAR operating at *f*_0_ needs to be designed for practical application; however, the substrate thickness may not equal an integer multiple of the half-wavelength due to the manufacturing and velocity (*ν*) tolerances of the substrate, etc., which will lead to the fabricated HBAR not resonating at *f*_0_. If the HBAR operating at *f*_0_ is needed, based on above study, the resonance frequency of a fabricated HBAR can be corrected to the designed frequency by modifying the substrate thickness.

**Figure 3:**
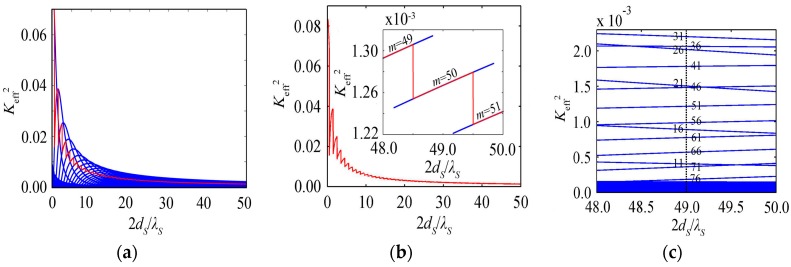
The HBAR consisting of ZnO_1.05μm_/Sapphire; (**a**) The variation curve of *K*_eff_^2^ with the substrate thickness; (**b**) The variation curve of *K*_eff_^2^ at the frequency closest to *f*_0_ with the substrate thickness; (**c**) The partial enlarged drawing of Figure 3a.

The behavior of *K*_eff_^2^ with the substrate thickness is composed of a series of intersecting blue lines shown in Figure 3a for the HBAR consisting of ZnO_1.05μm_/Sapphire. Each curve corresponding to a certain mode has a main peak and a series of lower peaks. With the increase of the number of modes, the main peak appears at the thicker substrate, and the corresponding peak value decreases gradually. As shown in the red line of Figure 3a or b, *K*_eff_^2^ at the resonance frequency closest to *f*_0_ declines rapidly and oscillates with the increase of the substrate thickness, and the oscillation amplitude gets smaller and smaller. Reference [18] demonstrates that the corresponding *Q* increases by adding to the substrate thickness; namely, it is at the expense of the lower *K*_eff_^2^ to increase the *Q* value, so the substrate thickness should be selected appropriately. *K*_eff_^2^ does not change monotonically with the increase of the number of modes for the HBAR with a certain substrate thickness, e.g., *K*_eff_^2^ has a maximum value at mode *m* = 31 when 2*d_S_*/*λ_S_* = 49, as shown in Figure 3c. Besides, the maximum value of *K*_eff_^2^ (*K*_eff_^2^_max_) declines rapidly with the substrate thickness increasing, as shown in the envelope of the intersecting curve family in Figure 3a.

### 3.2. K_eff_^2^ Varies with the Substrate Materials

**Figure 4:**
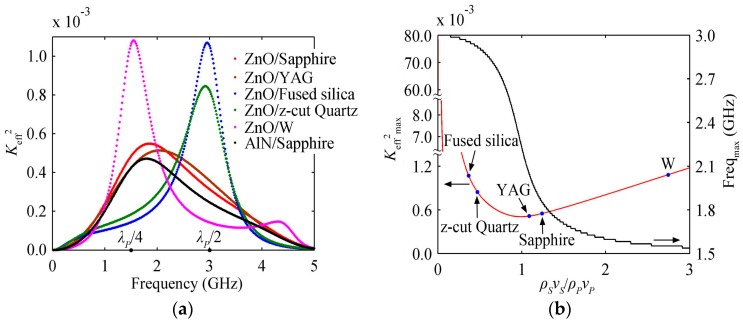
(**a**) The variation curve of *K*_eff_^2^ with resonance spectrum; (**b**) The variation curve of *K*_eff_^2^_max_ and Freq_max_ with $\rho_{s}\nu_{s}/\rho_{P}\nu_{P}$.

The behaviors of *K*_eff_^2^ with the resonance spectrum for the HBARs with several substrates, i.e., the HBARs with different characteristic impedance ratios of the substrate to piezoelectric film (*ρ_S_ν_S_*/*ρ_P_ν_P_*), are shown in Figure 4a, where the thickness of the ZnO and the substrate are a half-wavelength (1.05 μm) and 100 wavelengths at 3 GHz, respectively. It indicates that *K*_eff_^2^_max_ and the corresponding resonance frequency (Freq_max_) have a large difference for the HBARs with different *ρ_S_ν_S_*/*ρ_P_ν_P_*. Freq_max_ is close to the frequency where the piezoelectric film thickness is one-fourth of the wavelength for the hard substrate with a larger *ρ_S_ν_S_*/*ρ_P_ν_P_*, e.g., W, and it is close to the frequency where the piezoelectric film thickness is a half-wavelength for the soft substrate with a smaller *ρ_S_ν_S_*/*ρ_P_ν_P_*, e.g., fused silica. For a sapphire substrate with *ρ_S_ν_S_*/*ρ_P_ν_P_* = 1.24, the Freq_max_ equals 1.85 GHz and the corresponding piezoelectric film thickness is 0.31 wavelengths. Figure 4b shows that with the increase of *ρ_S_ν_S_*/*ρ_P_ν_P_*, *K*_eff_^2^_max_ decreases rapidly to a minimum value where *ρ_S_ν_S_*/*ρ_P_ν_P_* equals one firstly, then increases slowly. Fused silica or z-cut quartz as the substrate has a larger *K*_eff_^2^_max_ compared with sapphire or YAG, but a lower *Q* [18]. It is appropriate to choose sapphire or YAG as a substrate considering the effects of *K*_eff_^2^ on the function of the HBAR oscillators to be discussed later.

## 4. Relationship between the Effective Electromechanical Coupling Coefficient and the Piezoelectric Film

### 4.1. K_eff_^2^ Varies with the Piezoelectric Film Thickness

**Figure 5:**
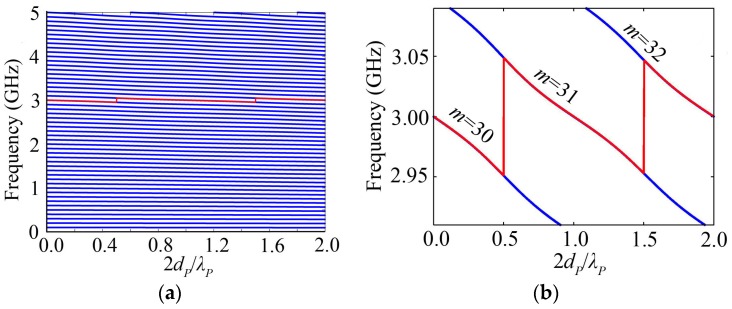
The HBAR consisting of ZnO/Sapphire_55.78μm_; (**a**) The variation curve of the resonance frequency with the piezoelectric film thickness; (**b**) The partial enlarged drawing of Figure 5a.

The blue lines of Figure 5a show the resonance spectrum changing with the piezoelectric film thickness for the HBAR consisting of ZnO/Sapphire_55.78μm_, where the substrate thickness corresponds to 15 wavelengths at 3 GHz. It can be seen that the HBAR has multi-frequency resonance characteristics similar to Figure 2a, but the change of the resonance spectrum is much smoother than that in the case of the substrate. Besides, the frequency of the same number of modes and SRF are smaller for thicker piezoelectric film. If the piezoelectric film thickness equals an integer multiple of the half-wavelength, the HBAR will resonate at *f*_0_ of 3 GHz; otherwise, the HBAR will not resonate at *f*_0_. The behavior of the resonance frequency closest to *f*_0_ with the piezoelectric film thickness is shown in the red line of Figure 5a or b, which is similar to the discussion in the case of the substrate, and its variations only contain three modes, namely *m* = 30, 31 and 32. With the increase of the piezoelectric film thickness, the resonance frequency closest to *f*_0_ jumps from *m* = 30 to *m* = 31 at 2*d_P_*/*λ_P_* of 0.5 and from *m* = 31 to *m* = 32 at 2*d_P_*/*λ_P_* of 1.5, respectively. The resonance frequency closest to *f*_0_ oscillates around *f*_0_, but its change amplitude is much smaller than that in the case of the substrate.

**Figure 6:**
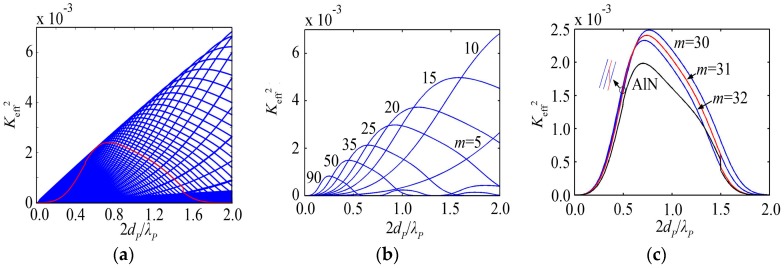
The HBAR consisting of ZnO/Sapphire_55.78μm_; (**a**) The variation curve of *K*_eff_^2^ with the piezoelectric film thickness; (**b**) The partial modes of HBAR shown in Figure 6a; (**c**) The partial enlarged drawing of Figure 6a close to mode *m* = 30.

The variation of *K*_eff_^2^ with the piezoelectric film thickness is composed of a series of intersecting blue lines shown in Figure 6a for the HBAR consisting of ZnO/Sapphire_55.78μm_. Each curve corresponding to a certain mode has a main peak, which is similar to the case of the substrate. As shown in Figure 6b, with the increase of the number of modes, the main peak appears at the thinner piezoelectric film, and the corresponding peak value decreases gradually. Besides, *K*_eff_^2^ reaches its maximum at a lower number of modes for the HBAR with a thicker piezoelectric film. As shown in the red line of Figure 6a or c, *K*_eff_^2^ at the resonance frequency closest to *f*_0_ has a maximum value when the piezoelectric film thickness 2*d_P_*/*λ_P_* ≈ 0.74, which is different from the case of the substrate.

### 4.2. K_eff_^2^ Varies with the Piezoelectric Film Materials

ZnO and AlN are commonly used piezoelectric film materials. Since ZnO has a larger *k_t_*^2^ than AlN according to Table 1, the HBAR with ZnO piezoelectric film has a larger *K*_eff_^2^ at the same number of modes, normalized thickness or frequency, as shown in Figure 6c and Figure 4a. Besides, due to the large extensional wave velocity *ν* of AlN (see Table 1), AlN piezoelectric film is much thicker than ZnO piezoelectric film when operating at the same frequency; however, the much thinner piezoelectric film may lead to short-circuit caused by pinholes in the process of sputtering, which will result in a lower yield. Therefore, it is more appropriate to use AlN as the piezoelectric film when the HBAR operates at a higher frequency.

## 5. Influence of the Electrode on the Effective Electromechanical Coupling Coefficient

Since the electrode thickness is comparable with the piezoelectric film thickness, the influence of the electrode should be taken into account for the HBARs operating at gigahertz frequencies, and Au and Al are two commonly used electrode materials. The HBARs discussed in the following have the same thickness of the top and bottom electrodes, and the sandwich transducer composed of electrode/ZnO/electrode operates at 3 GHz.

### 5.1. K_eff_^2^ Varies with the Substrate Thickness Considering the Effect of the Electrode

**Figure 7:**
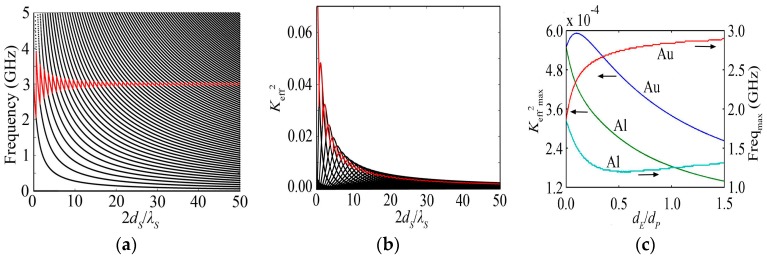
(**a**) The variation curve of the resonance frequency with the substrate thickness for the HBAR consisting of Au/ZnO/Au/Sapphire; (**b**) The variation curve of *K*_eff_^2^ with the substrate thickness for the HBAR consisting of Au/ZnO/Au/Sapphire; (**c**) The variation curve of *K*_eff_^2^_max_ with *d_E_*/*d_P_* for the HBAR consisting of Au(Al)/ZnO/Au(Al)/Sapphire with the substrate thickness 2*d_S_*/*λ_S_* of 200, where the blue and red lines represent *K*_eff_^2^_max_ and Freq_max_ of Au, respectively, and the green and light blue lines represent *K*_eff_^2^_max_ and Freq_max_ of Al, respectively.

For the HBAR consisting of Au/ZnO/Au/Sapphire with the thickness ratio of the electrode to the piezoelectric film (*d_E_*/*d_P_*) of 0.1884, the variations of the resonance spectrum and *K*_eff_^2^ with the substrate thickness are shown in the black lines of Figure 7a,b, respectively, which are similar with the case ignoring the effect of the thin electrode shown in the blue lines of Figure 2a and Figure 3a. The oscillation behaviors of the resonance frequency closest to 3 GHz and the corresponding *K*_eff_^2^ with the substrate thickness, as shown in red line of Figure 7a,b, are also similar with the case shown in red line of Figure 2a and Figure 3a, respectively.

### 5.2. The Relationship between K_eff_^2^ and the Electrode Thickness and Materials

For the HBAR consisting of Au/ZnO/Au/Sapphire with 2*d_S_*/*λ_S_* of 200, the *K*_eff_^2^_max_ and Freq_max_ changing with *d_E_*/*d_P_* are shown in Figure 7c. It can be seen that *K*_eff_^2^_max_ has a maximum value at *d_E_*/*d_P_* of 0.0958, which is 7.92% larger than that in the case ignoring the effect of the thin electrode; meanwhile, Freq_max_ has an increase of 27.46%. For the Al electrode, *K*_eff_^2^_max_ declines rapidly with the increase of *d_E_*/*d_P_*, and it has less value than that in the case of Au. Freq_max_ decreases rapidly and then increases slowly with the increase of *d_E_*/*d_P_*. The proper *d_E_*/*d_P_* is required for the HBAR because the thicker electrode will reduce the *K*_eff_^2^_max_, and the thinner electrode will affect the *Q* value due to the ohmic resistance increasing. Therefore, it is better to use Au as the electrode than Al, and *d_E_*/*d_P_* = 0.10–0.26 is appropriate.

## 6. Experiment

In this paper, a HBAR based on a sapphire substrate was fabricated. The 400-μm-thick *c*-axis sapphire polished on both sides was used as the substrate. Au thin film was deposited upon the substrate and then patterned to form the bottom electrode of the piezoelectric transducer. ZnO piezoelectric film was deposited on the bottom electrode by magnetron sputtering and patterned to reveal the contact pads on the bottom electrode. The top electrode of Al was then deposited and patterned. The structure of the HBAR is Al_100nm_/ZnO_0.6μm_/Au_100nm_/Sapphire_400μm_ and it has a square active area of 100 μm × 100 μm.

**Figure 8:**
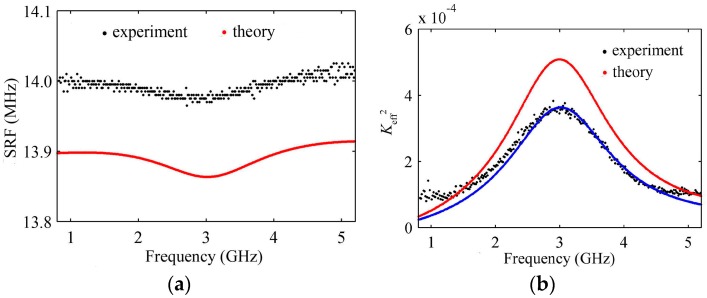
(**a**) The variation curve of SRF with the resonance frequency; (**b**) The variation curve of *K*_eff_^2^ with the resonance frequency.

The HBAR was measured by the Agilent E5071C network analyzer (Agilent Technologies Inc., Palo Alto, CA, USA). The behaviors of the SRF with the frequency are shown in Figure 8a; the experimental and theoretical results have the same variation trends, and the experimental results are 0.7% larger than the theoretical results, which may be caused by the difference in characteristic parameters (*ρ*, *ν*) between the sputtered thin film and single-crystal materials listed in Table 1, the measuring errors of each layer’s thickness, etc. The experimental values of *K*_eff_^2^ are different from the theoretical results (see Figure 8b), and the main reason may be that the electromechanical coupling coefficient *k_t_*^2^ of sputtered piezoelectric film is less than that of the single-crystal material listed in Table 1 [19]. The experimental curve fits quite well with the theoretical result if *k_t_*^2^ is decreased by 27%, ignoring the other factors, as shown in the blue dots of Figure 8b.

## 7. Effects of *K*_eff_^2^ on the Function of the Crystal Oscillators Constructed with HBARs

*K*_eff_^2^ is directly related to the bandwidth and insertion loss in the filters [20], and for a larger *K*_eff_^2^, the bandwidth is larger at the same insertion loss or the insertion loss is smaller at the same bandwidth. For a HBAR, *K*_eff_^2^ seems to not be an influencing factor of the oscillators’ function since it operates at a single frequency, but this was not the case in reality. In order to generate oscillation, two conditions must be satisfied for the crystal oscillators constructed with HBARs [21]: the amplifier must be capable of providing a loop gain of one and the oscillators satisfies the following equation,

(8) $$C_{0}+C_{x}\leq Q_{M}\frac{N^{2}C}{2}$$

where *C_x_* is the load capacitance in parallel with the HBAR in the oscillators and *Q_M_* is the mechanical quality factor (when the mechanical loss of the HBAR is taken into account, the mechanical branch of Figure 1c adds a mechanical resistance *R*, and *Q_M_* = *ωL*/*R* = 1/*ωCR* [18]) of the HBAR.

Substitute $N^{2}C=\frac{8}{\pi^{2}}{K_{\mathrm{eff}}}^{2}C_{0}$ obtained from Equations (7) into (8), and the crystal oscillators generating oscillation must satisfy,

(9) $${K_{\mathrm{eff}}}^{2}\cdot Q_{M}\geq \frac{\pi^{2}}{4}\left(\frac{C_{x}}{C_{0}}+1\right)$$

**Figure 9:**
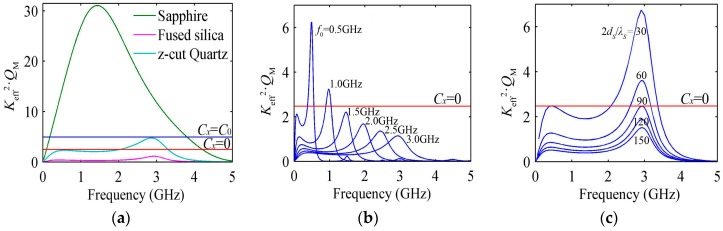
The variation curve of *K*_eff_*^2^*·*Q_M_* with the resonance frequency; (**a**) The HBARs consisting of ZnO/substrate; (**b**) The different operating frequency using fused silica as substrate; (**c**) The different thickness of fused silica substrate.

The behavior of *K*_eff_^2^·*Q_M_* with the frequency is shown in Figure 9a for the HBARs consisting of ZnO/substrate (sapphire, fused silica and z-cut quartz), where the thickness of the piezoelectric film and substrate are a half-wavelength and 100 wavelengths at 3 GHz, respectively. The lowest requirement for the HBARs’ oscillation is that there are some *K*_eff_^2^·*Q_M_* values above the red line, namely the case that the load capacitance *C_x_* equals zero in Equation (9). The HBAR with the fused silica substrate is not suitable as the crystal oscillators since its *K*_eff_^2^·*Q_M_* curve is below the red line. For the HBAR with the z-cut quartz substrate, there are 87 modes satisfying their *K*_eff_^2^·*Q_M_* values above the red line within a relatively narrow frequency range. However, the HBAR with the sapphire substrate can be applied as the crystal oscillators within a very wide frequency range (including 276 modes). When the load capacitance *C_x_* equals *C*_0_, only the HBAR with the sapphire substrate can be used as the crystal oscillators (including 242 modes). If the lower operating frequency *f*_0_ (*Q*_M_ is inversely proportional to the frequency) or the thinner substrate (*K*_eff_^2^ is larger for the thinner substrate) is selected, the HBARs with the fused silica substrate can also be used as the crystal oscillators, as shown in Figure 9b,c. Therefore, the HBARs with a larger *K*_eff_^2^ or, strictly speaking, a larger *K*_eff_^2^·*Q_M_* are required for the crystal oscillators.

## 8. Conclusions

The influences of the material and thickness properties of the different layers composing a HBAR on the resonance spectrum characteristics of *K*_eff_^2^ have been studied, and the effects of the common substrate, electrode and piezoelectric materials as well as their thicknesses have been evaluated. Based on this study, the structure of Au/ZnO/Au/Sapphire (or YAG) with the appropriate thickness of the substrate, piezoelectric film and electrode should be selected, as this can make the crystal oscillators obtain a larger frequency stability and frequency hopping bandwidth due to its larger *K*_eff_^2^·*Q_M_* value. When the HBARs with the fused silica substrate operate at a low frequency or have a thin substrate, they can also be used as the crystal oscillators. Besides, the AlN piezoelectric film with a high velocity can be used for higher frequency, even though it has a smaller *K*_eff_^2^ than ZnO.

## Figures and Tables

**Table 1:** Material parameters of composite resonators used.

Structure	Material	*ν* (m/s)	*ρ* (kg/m^3^)	*k*_t_^2^	*ρν* (×10^7^ kg·m^−2^·s^−1^)
Piezoelectric film	ZnO	6301	5680	0.0784	3.5790
	AlN	10,400	3260	0.065	3.3904
Substrate	Fused silica	5973	2200	-	1.3141
	z-cut Quartz	6359	2651	-	1.6858
	Yttrium aluminum garnet (YAG)	8558	4550	-	3.8939
	Sapphire	11,155	3986	-	4.4464
	Tungsten (W) ^1^	5113	19,200	-	9.8170
Electrode	Al	6330	2695	-	1.7059
	Au	3104	19,300	-	5.9907

^1^ W is not the commonly used material and is used in the analysis due to its high characteristic impedance.
